# Performance of HRP-2 based rapid diagnostic test for malaria and its variation with age in an area of intense malaria transmission in southern tanzania

**DOI:** 10.1186/1475-2875-9-294

**Published:** 2010-10-26

**Authors:** Anne Laurent, Joanna Schellenberg, Kizito Shirima, Sosthenes C Ketende, Pedro L Alonso, Hassan Mshinda, Marcel Tanner, David Schellenberg

**Affiliations:** 1 Faculty of Infectious and Tropical Diseases, London School of Hygiene & Tropical Medicine, London, UK; 2 Ifakara Health Institute, Dar es Salaam, Tanzania; 3 Barcelona Centre for International Health Research, Barcelona, Spain; 4 Swiss Tropical and Public Health Institute, P.O. Box 4002 Basel, Switzerland; 5 University of Basel, 4002 Basel, Switzerland

## Abstract

**Background:**

The use of malaria rapid diagnostic tests (RDTs) has been widely advocated to improve *Plasmodium falciparum *diagnosis, especially in settings where quality microscopy is not available. RDTs based on the detection of histidine-rich protein 2 (HRP-2) can remain positive for several weeks after an infection is cured, due to the persistence of HRP-2 antigens. As a result, test specificity may vary between age groups with different prevalence of *P*. *falciparum *infection.

**Methods:**

A community-based cross-sectional survey, carried out in southern Tanzania in July and August 2004, evaluated the performance of the Paracheck Pf in comparison with microscopy (number of *P. falciparum *parasites/200 leucocytes). A sample of 598 individuals living in an area of intense malaria transmission had demographic data collected before an RDT was performed. HRP-2 test sensitivity, specificity, positive and negative predictive values were calculated and compared between distinct age groups, using microscopy as "gold standard".

**Results:**

The overall malaria prevalence was 34.3% according to microscopy and 57.2% according to the HRP-2 test. The HRP-2 test had a sensitivity of 96.1%, a specificity of 63.1%, a positive predictive value of 57.6% and a negative predictive value of 96.9%. The test sensitivity was higher (ranging from 98% to 100%) amongst people less than 25 years of age, but decreased to 81.3% in older adults. The HRP-2 test specificity varied between age groups, ranging from 25% among children of five to nine years of age, to 73% among adults aged 25 or more. The test positive predictive value increased with malaria prevalence, while the negative predictive value was consistently high across age groups.

**Conclusions:**

These results suggest that the performance of HRP-2 tests in areas of intense malaria transmission varies by age and the prevalence of *P. falciparum *infection. The particularly low specificity among children will lead to the over-estimation of malaria infection prevalence in this group.

## Background

Rapid diagnostic tests (RDTs) for malaria were developed in the early 1990's, and welcomed with great enthusiasm as a tool to improve malaria diagnosis, especially in remote areas of Africa where access to microscopy is often limited due to the lack of resources and skilled technicians. In such settings, malaria diagnosis frequently relies on clinical criteria, which have a low specificity, leading to overuse of malaria drugs and potentially exerting increased drug pressure on malaria parasite populations leading to the selection of drug-resistant parasites. The urgent need to deploy affordable and accurate RDTs grew with the emergence of widespread resistance of *P. falciparum *to chloroquine and sulphadoxine pyrimethamine (SP). As a result, malaria treatment increasingly relies on artemisinin combination therapy (ACT), a drug regimen that is only cost-effective if it is targeted exclusively at patients with a confirmed malaria diagnosis [1]. One RDT type is based on the detection of histidine-rich protein 2 (HRP-2), a water-soluble protein produced by asexual forms and young gametocytes of *P*. *falciparum*. HRP-2 persists after cure of active infections, potentially leading to false-positive test results after treatment.

According to the World Health Organization's (WHO) recommendations, RDTs should demonstrate a minimum sensitivity of 95% (100% when parasite density is above 100/μL), and a minimum specificity of 90% [2]. HRP-2 based assays have been tested in a wide range of clinical settings and endemicity sites, though only recently in a standardized and co-ordinated way [3]. In areas of low endemicity, where the vast majority of infections are symptomatic and affect all age groups equally, HRP-2 based tests have been shown to have satisfactory specificity, according to WHO standards [4-6]. Conversely, when transmission is intense and perennial, and the prevalence of parasitaemia in the population is high, the tests have shown less consistent performance results and might yield a large number of false positive results. Table 1 presents HRP-2 test performance estimates compared to microscopy in *P. falciparum *highly endemic areas as reported in previous studies [7-12]. With the exception of one study, the test specificity is consistently inferior to the minimum required of 90%. Many studies have demonstrated that the sensitivity of HRP-2 based tests declines sharply when the parasite density falls below 100 to 500 parasites/μL [6,8,9,13-15].

**Table 1:** Studies of HRP-2 performance compared to microscopy, in *P. falciparum *endemic areas.

*Reference*	*Location*	*Study population*	*Transmission (prevalence of positive blood smear in the study population)*	*Sensitivity*	*Specificity*
Beadle [8]	Kenya	Children aged 9 to 14 years	Hyperendemic (86%)	87%	88%

Swathout[11]	Democratic Republic of Congo	Symptomatic children aged 6-59 months	Highly endemic and seasonal	100%	52%

Huong [9]	Vietnam	Symptomatic and control individuals aged 4-60 years	Hyperendemic	96%	100%

Guthmann [7]	Uganda	Symptomatic individuals	Hyperendemic (57%)	97%	88%

Williams [12]	Tanzania	Symptomatic individuals of all age	Hyperendemic (32%)	94%	89%

Shiff [10]	Tanzania	na	Holoendemic	89%	88%

na = not applicable

A relatively small amount of research has been carried out to investigate how the specificity of HRP-2 based tests, with microscopy as the "gold standard", varies between areas of distinct endemicity and malaria prevalence. Their findings are summarized in Table 2, and show a consistent decrease of the test specificity with increasing prevalence of *P. falciparum *infection. This is despite the short-comings of microscopy, the performance of which varies according to the way in which the slide is read [16]. Hopkins *et al *[17] suggested that a proportion of HRP-2 false positive results may have been the result of sub-patent parasitaemia undetectable by microscopy and reported a change of overall specificity in the whole study population from 71% to 88% when the microscopy results were corrected by polymerase chain reaction (PCR) analysis. The persistence of HRP-2 protein in the blood several weeks after parasite clearance is known to limit the potential usefulness of the test in highly endemic areas, as positive results might be the result of recent treated infections [11,14].

**Table 2:** Studies comparing HRP-2 performance between areas of different *P. falciparum *endemicity, using microscopy as a "gold standard".

*Reference*	*Setting*	*Prevalence of positive blood smears in the study population (level of malaria transmission as reported by the author)*	*Sensitivity*	*Specificity*
Mharakurwa [24]	Zimbabwe	10% (hypoendemic)	91%	92%
		40% (hyperendemic)	95%	85%
		59% (mesoendemic)	93%	72%

Hopkins [17]	Uganda	4% (hypoendemic)	na	98%
		33 to 85% (hyperendemic)	na	55%

Mboera [14]	Tanzania	4% (unstable, prone to outbreaks)	92%	99%
		14% (unstable, prone to outbreaks)	97%	97%
		22% (unstable, prone to outbreaks)	84%	91%
		49% (unstable, prone to outbreaks)	98%	90%
		46% (perennial)	81%	95%

Abeku [25]	Tanzania	1% (hypoendemic)	90%	100%
		49% (mesoendemic)	91%	65%

na = not applicable

Note: The studies examined different volumes of blood before declaring a slide negative: studies [26,12] examined 100 fields and study [9] 200 fields before declaring a slide negative; study [22] declared slides negative if no parasite was seen by the time 400 white blood cells counted.

The study presented here was carried out in an area of intense and perennial malaria transmission in Tanzania. In such transmission settings, the prevalence of malaria infection varies with age, and so the performance, especially the specificity, of HRP-2 based tests, might vary between distinct age groups. This community-based study aims to estimate and compare the sensitivity, specificity, positive predictive value (PPV) and negative predictive values (NPV) of an HRP-2 based assay between age groups, using microscopic examination of blood smears as the "gold standard".

## Methods

### Study area

This study was nested within a household baseline survey carried out in 2004 in the Tandahimba district of Mtwara region southern Tanzania. The study area and survey are described in more detail elsewhere [18]. Malaria cases present to health facilities all year round and parasitaemia prevalence in the community rises sharply over the first few months of life, consistent with intense malaria transmission. *Plasmodium falciparum *is the main species responsible for malaria in Tanzania, with *Plasmodium malariae *and *Plasmodium ovale *occurring in less than 5% of infections, and usually mixed with *P. falciparum. Plasmodium vivax *is only occasionally found. The country's national strategy against malaria at the time of the survey relied on early treatment of episodes with sulphadoxine/pyrimethamine (SP) as the first-line drug and amodiaquine as second-line drug, and the promotion of insecticide-treated bed nets to reduce exposure.

### Sampling method

Eight clusters of 30 households were sampled from sub villages of Litehu and Namikupa divisions of Tandahimba district using a modified EPI-type sampling scheme, such that each household in a division had an equal chance of being included in the sample [18]. A sequential series of 649 individuals of all ages constituted our study sample and had a microscopic examination of a blood smear performed in a research laboratory with quality assurance procedures.

### Data collection

This cross-sectional survey was carried out during July and August 2004 by two groups of seven experienced field workers accompanied by a supervisor. Villages were visited one day before the survey, and an invitation letter left in each of the selected households. A modular questionnaire was used to collect data on age, sex, location of household, demographic characteristics and various health-related information. All household members were invited for a malaria check by RDT and a small quantity of blood was collected on a filter paper. The teams collected, stained and stored blood slides for subsequent microscopic examination.

### Laboratory techniques

The HRP-2 test used to detect *P. falciparum *parasitaemia was the Paracheck rapid diagnostic test device (Orchid Biomedical Systems, Goa, India). A Swahili version of the technical sheet was developed to provide the details of the testing procedure for the field workers. A training session was conducted by an experienced laboratory technician before a practice session in the classroom, followed by a supervised pilot of all procedures in the field. Blood films were prepared by experienced laboratory assistants and labelled with a unique sample identification number before transportation to the research laboratory where they were stained with Giemsa and the number of asexual and sexual *P. falciparum *parasites per 200 leucocytes was counted. All blood slides were read twice by two independent technicians, and the results compared. A third reading was performed in case of positive-negative or species disagreement, or if the ratio of two positive results was greater than 1.5 or less than 0.67 with an absolute difference in parasite density of less than 30 parasites/μl. The definitive parasite count was then calculated assuming a white blood cell count of 8,000/μL.

### Data processing and analysis

Data were entered into hand held computers at the point of data collection [19]. Standard range and consistency checks were performed at the time of data entry. Data collected via the questionnaire and relevant to the study question were extracted and linked to the laboratory results through the individual identification numbers. Complete microscopy, RDT and demographic data were available for 598 individuals. The microscopic examination of blood slides revealed the presence of gametocytes in nine of these blood samples, all of which also had asexual forms.

After each linkage, a consistency check was performed for the variables common to the various data sets. Data were analyzed using Stata (version 8, College Station, Texas, USA), according to a pre-defined analytical plan. The study sample was divided into four age groups on the basis of prevalence of parasitaemia: 0 to 4 years, 5 to 9 years, 10 to 24 years, and 25 years and above. Three categories of parasite densities density were created among individuals with a positive blood smear, with cut-off points at 500 and 5,000 parasites/μL.

Malaria prevalence was calculated in each category of age, sex and geographical location, and reported with a 95% confidence interval. Using microscopy as "gold standard", each HRP-2 result was categorized as true positive (TP), true negative (TN), false positive (FP) or false negative (FN), so that sensitivity (TP/TP+FN), specificity (TN/TN+FP), positive predictive value (TP/TP+FP) and negative predictive value (TN/TN+FN) could be calculated in each age group, with their 95% confidence interval. The test performance was also evaluated for categories of parasite density. In accordance with the study design, all measurement of malaria prevalence and HRP-2 test performance were performed using the "svy" command in STATA, in order to account for the sampling strategy. Geometric means of parasite densities of positive blood slides between age groups were computed on a logarithmic scale, back-transformed and compared using a one-way analysis of variance (ANOVA).

Proportions were compared using Fisher's exact test, and tests for trend were performed using linear regression. A logistic regression model was used to assess the variation of test sensitivity between age groups, adjusting for parasite density. A P-value < 0.05 was considered statistically significant.

### Ethical aspects and informed consent

An information sheet about the study was drawn up in Swahili, and read out to each household head before written informed consent was sought. In the event of a positive HRP-2 test result, study participants were offered treatment, according to the national guidelines. This study was approved by the ethics committees of the Ifakara Health Institute (formerly Ifakara Health Research and Development Centre), the Tanzanian National Medical Research Co-ordinating Committee, the London School of Hygiene and Tropical Medicine and the Swiss Tropical& Public Health Institute (formerly Swiss Tropical Institute).

## Results

### Baseline characteristics of the sample and *P. falciparum *infection prevalence

**Figure 1:**
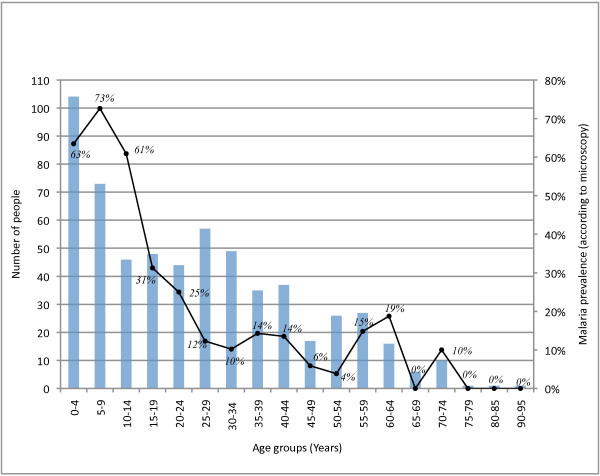
**Sample age distribution and malaria prevalence by 5-year age groups**. Number of individuals in each 5-year age group (blue columns). Malaria prevalence according to microscopy in each 5-year age group (black line).

Sampled individuals were equally distributed across two divisions (Litehu and Namikupa) of Tandahimba District. Each of those two divisions is divided into three wards, where the sample population was approximately equally distributed (Table 3). Among the 598 individuals, 45 were children under two years of age and 543 were aged two years or more. Microscopy identified *P. falciparum *infections in 205 of the 598 blood smears. The sample age distribution is presented in Figure 1, along with malaria prevalence according to microscopy by five-year age groups.

**Table 3:** *Plasmodium falciparum *infection prevalence by baseline characteristics, according to blood smear microscopic examination and HRP-2 test.

		Microscopy			Rapid diagnostic test		
	No of observations	Prevalence (%)	95% CI	P-value (†)	Prevalence (%)	95% CI	P-value (†)
**Sex**				0.18			0.54
Female	320	31.3	[23.3-40.5]		56.3	[46.2-65.8]	
Male	278	37.8	[28.2-48.5]		58.1	[44.0-71.3]	

**Geographical location**							
***Division***				0.40			0.93
TAN Litehu	303	31.7	[20.4-45.7]		56.8	[37.0-74.6]	
TAN Namikupa	295	37.0	[30.9-43.5]		57.6	[46.6-67.9]	

***Ward***				0.029			< 0.001
Mkoreha	97	42.3	[32.3-52.3]		67.0	[57.5-76.5]	
Lyenje	109	38.5	[29.2-47.8]		70.6	[62.0-79.3]	
Mihambwe	106	35.8	[26.6-45.1]		54.7	[45.1-64.4]	
Chaume	99	35.4	[25.8-44.9]		56.6	[46.6-66.5]	
Kitama	92	32.6	[22.8-42.4]		51.1	[40.7-61.5]	
Mkonjowano	95	20.0	[11.8-28.2]		41.1	[31.0-51.1]	

**Age**				<0.001			<0.001
0 to 4 years	104	63.5	[49.4-75.5]		76.9	[64.1-86.2]	
5 to 9 years	73	72.6	[54.2-85.6]		91.8	[82.3-96.4]	
10 to 24 years	138	39.1	[26.8-53.1]		73.2	[54.3-86.2]	
>= 25 years	283	11.3	[7.8-16.1]		33.2	[24.0-44.0]	

**Total**	**598**	**34.3**	**[26.5-42.0]**		**57.2**	**[45.9-68.5]**	

(^†^) Fisher's exact test

The HRP-test yielded positive results for 342 out of the 598 blood specimens (57.2%, [95% CI 45.9-68.5%]. *Plasmodium falciparum *infection prevalence based on microscopy and HRP-2 test, by baseline characteristics, is presented in Table 3. According to microscopy, 34.3% [95% CI 26.5-42.0%] of the study sample was infected with *P. falciparum *and no significant differences were found between genders or across divisions (Table 3). The malaria prevalence varied between wards, ranging from 20% in Mkonjowano to 42.3% in Mkoreha (p < 0.001). The prevalence increased in childhood, from 64% in children below the age of five, to 73% in children aged five to nine years, before decreasing with increasing age in older children and adults. The difference of malaria prevalence between age categories was statistically significant (p < 0.001).

### HRP-2 test performance

**Figure 2:**
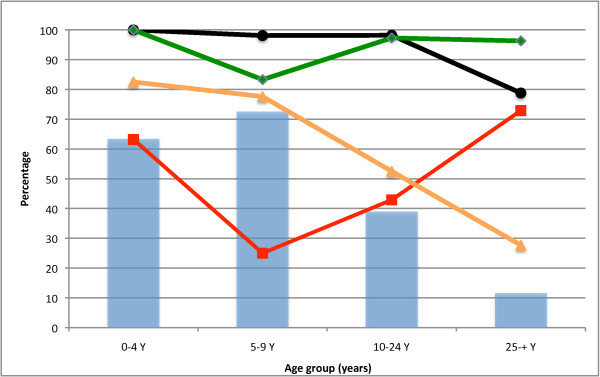
**HRP-2 test performance by 5-year age group**. HRP-2 test sensitivity (black circles), specificity (red squares), positive predictive value (orange triangles) and negative predictive value (green diamonds) by 5-year age group. Malaria prevalence according to microscopy (blue bars).

The HRP-2 test performance results by age group are summarized in Additional file 1, and graphically illustrated in Figure 2. Overall, the HRP-2 based test demonstrated a sensitivity of 96.1% [95% CI 92.5-98.3%], a specificity of 63.1% [95% CI 58.1-67.9%], a positive predictive value (PPV) of 57.6% [95% CI 52.2-62.9%] and a negative predictive value (NPV) of 96.9% [95% CI 93.9-98.6%].

The HRP-2 test *sensitivity *was consistently high among children and young adults, but decreased to 81% in adults above 25 years of age. HRP-2 test *specificity *varied dramatically with age, from 25% among individuals of five to nine years of age, to 73% among individuals aged 25 years or more. As the prevalence of malaria infection differed between age groups, the test specificity appeared to decrease with increasing malaria prevalence. The *PPV *of the HRP-2 test decreased as age increased, while its *NPV*, on the contrary, remained steady at a high value across age groups.

No evidence of a difference in sensitivity across categories of parasite density was found (Table 4). Children below five years of age had the highest parasitaemia, and the density decreased significantly as age increased (P < 0.001) (Table 5). However, parasite densities were generally low. Adjustment for parasite density did not alter the association between test performance and age groups. Among the eight false negative HRP-2 test results, six had a parasite density less than 500 parasites/μL, while the two others had a parasite density of 2,500 and 10,500 parasites/μL. The majority of false negative results (six out of eight) were individuals belonging to the older age group.

**Table 4:** HRP-2 test sensitivity by category of parasite density

No of parasites/μL	No of observation	Sensitivity	95% CI
<500	81	92.6%	[78.0-97.8]
500-4999	74	100%	-
>5000	50	96.0%	[81.7-99.2]

P value (Fisher's exact test) 0.13

**Table 5:** Mean parasite density by age group among individuals with a positive blood slide

		Parasite density	
Age category (years)	No of observation	Parasites/μL(*)	95% CI
0-4	66	3222	[2196-4726]
5-9	53	1349	[864-2106]
10-24	54	512	[372-706]
25-+	32	335	[206-547]

Total	205	1118	[887-1408]

P value (Anova test) < 0.001

(*) Geometric means of parasites density in positive slides

## Discussion

This paper has evaluated HRP-2 test performance in people of distinct age groups living in an area of intense *P. falciparum *transmission. The age pattern of parasitaemia and the high prevalence of *P. falciparum *infection in the childhood population indicate the intense malaria transmission in our study area.

HRP-2 test *sensitivity *was consistently high in the younger age groups, in keeping with the findings of previous studies in similar settings [7,9,11,12]. There was no evidence that the lower sensitivity in people aged 25 years or more was due to lower parasite density in older people. Numerous studies have shown that HRP-2 tests might fail to detect low-level parasitaemia [6,8,9,13-15]. In the current study, two cases of *P. falciparum *infection with high parasite density were undetected by the test. While similar reports exist [20-22], the cause has not been elucidated. It has been suggested that the HRP-2 test sensitivity could be affected by the variability of the target antigen in specific settings [23] but this hypothesis is unlikely to explain the findings in the current study as antigenic polymorphism is geographically based. An alternative hypothesis is that age dependent immune status might lower HRP-2 test sensitivity, independently of parasite density [15].

With respect to the HRP-2 test *specificity*, over one third of the individuals with a positive HRP-2 test result had a negative blood slide. The overall HRP-2 test specificity in our study is substantially lower than that found by all but one previous study sharing a similar methodology in similar malaria transmission settings [7-12,14,17,24]. In addition, the current study found that the test specificity varied with age, and was inversely related to the prevalence of positive blood slides in different age groups, i.e. the specificity decreased as malaria prevalence increased. These findings corroborate the results of a recent study where the investigators found an overall specificity of 65%, and a decrease in specificity with decreasing age and increasing malaria prevalence in symptomatic patients [25]. Another study reported similarly low HRP-2 test specificity (52%) in symptomatic children under five years of age in an area of intense transmission [11].

These striking findings have to be interpreted with some caution as this difference may have arisen for various methodological reasons. Although the microscopic examination of blood slides was subject to quality control, its use as "gold standard" might have been problematic, as it does not have perfect sensitivity. In areas of intense malaria transmission, a high proportion of the population carries low level of parasites at any given time. It is well established that PCR has a sensitivity superior to microscopy when the parasite density is low [26]. Previous studies suggest that a proportion of false positive HRP-2 results are due to sub-patent parasitaemia undetectable by microscopy [17,27].

The vast majority of comparable published studies on HRP-2 performance using microscopy as a "gold standard" in areas of intense malaria transmission recruited symptomatic participants at health facilities. The samples in this study were from mainly asymptomatic participants. Therefore, it is possible that the proportion of individuals with sub-patent parasitaemia was higher than in other studies. This hypothesis is also supported by the relatively low mean parasite density in this study's sample.

It has been reported that the RDT can stay positive for more than five weeks after successful treatment, because of prolonged persistence of HRP-2 antigen in the circulation after parasite death [11]. Information on recent past anti-malarial treatment was not elicited for the majority of the study population, and a high proportion of false positives may have been the result of recent treated infections, and these are likely to have been more frequent in younger age groups. This effect may be exaggerated if enhanced immunity acquired with age hastens the clearance of HRP-2 antigens. In this respect, rapid diagnostic tests based on detection of the parasite's lactate dehydrogenase (pLDH) are less likely to suffer the variable specificities in different age groups that we document because p-LDH is cleared from the circulation rapidly on effective treatment of infection.

HRP-2 tests are attractive because they are rapid and relatively easy to perform, and do not require equipment or electricity. However, our results suggest that their interpretation needs caution. As the risk of malaria infection depends mostly on the intensity of malaria transmission, the use of protective measures against infection and age-dependent immunity against *P*. *falciparum*, it seems reasonable to deduce that the different age groups in our study reflect distinct categories of likelihood of infection. As the HRP-2 assay's positive predictive value varies widely with the likelihood of current or recent infection at the time the test is performed, its use for the comparison of malaria prevalence between different age groups in a given malaria transmission setting, or for the comparison of malaria prevalence between populations living in different transmission settings, might be misleading. In populations with a high likelihood of infection, in whom test specificity is expected to be relatively low, the use of an HRP-2 test to evaluate the impact of a protective intervention will tend to under-estimate the effect of the intervention. Such comparisons may be more robust if based on blood slide or molecular data.

When HRP-2 tests are used for primary diagnosis in area of intense transmission, our results suggest a risk of malaria over-diagnosis in children, which might have serious consequences as the diagnosis of non-malaria febrile illnesses might be missed or delayed. This concern may not be so important if the variation in specificity with age is less marked in symptomatic individuals, who will tend to have a higher parasitaemia, but warrants further investigation. In practice, clinicians should bear in mind that their therapeutic and case-management decisions should not exclusively be based on the HRP-2 test result but should take into consideration the age of the patient, the clinical presentation (suggesting a non-malaria pathology) and a detailed history of recent drug treatment.

## Conclusions

The relevance of HRP-2 based assay results depends on the malaria transmission profile, the age of the patient and the aim of testing. HRP-2 test users should be aware of its very low specificity in some population age groups and malaria transmission settings, when compared with microscopy. HRP-2 test performance against PCR should be investigated in different transmission settings to determine the proportion of false positive results due to sub-patent parasitaemia. Comparison against PCR is likely to reduce variation in specificity but strong theoretical grounds remain to expect that the phenomenon of variation in test performance in different age groups and transmission settings will remain.

## List of abbreviations

ACT: Artemisinin Combination Therapy; HRP-2: Histidine Rich Protein 2; NPV: Negative Predictive Value; PCR: Polymerase Chain Reaction; PPV: Positive Predictive Value; RDT: Rapid Diagnostic Test; SP: Sulphadoxine-Pyrimethamine; WHO: World Health Organization.

## Competing interests

The authors declare that they have no competing interests.

## Authors' contributions

The study was conceived & designed by DS, JAS, PLA, HM and MT. Substantial contributions to acquisition of the data were made by KS. Initial analysis and interpretation of the data was done by AL, SK, JAS and DS. The manuscript was drafted by AL, JAS and DS. All authors were involved in critical revision for important intellectual content and approved the final version of the manuscript.

## Supplementary Material

Additional file 1 **HRP-2 test performance by age groups and categories of likelihood of malaria infection**. (†) Fisher's exact test. (±) Test for a linear trend. Click here for file
