# Supplementary material for: Performance of HRP-2 based rapid diagnostic test for malaria and its variation with age in an area of intense malaria transmission in southern tanzania
Source: Malar J. 2010 Oct 26;9:294. doi: 10.1186/1475-2875-9-294 (PMC2974751; doi:10.1186/1475-2875-9-294)
Supplement: Additional file 1 — HRP-2 test performance by age groups and categories of likelihood of malaria infection. (†) Fisher's exact test. (±) Test for a linear trend. [file 1475-2875-9-294-S1.DOC]

|  | Number of observations | Malaria prevalence (%) | Sensitivity | | Specificity | | Positive  predictive value | | Negative predictive value | |
| --- | --- | --- | --- | --- | --- | --- | --- | --- | --- | --- |
|  |  |  | % | 95% CI | % | 95% CI | % | 95% CI | % | 95% CI |
| Overall | 598 | 34.3 | 96.1 | [92.5-98.3] | 63.1 | [58.1-67.9] | 57.6 | [52.2-62.9] | 96.9 | [93.9-98.6] |
| Age |  |  |  |  |  |  |  |  |  |  |
| 0-4 | 104 | 63.5 | 100 | [89.9-100] | 63.2 | [46.0-78.2] | 82.5 | [72.4-90.1] | 100 | [85.8-100] |
| 5-9 | 73 | 72.6 | 98.1 | [89.9-100] | 25.0 | [8.7-49.1] | 77.6 | [65.8-86.9] | 83.3 | [35.9-99.6] |
| 10-24 | 138 | 39.1 | 98.1 | [90.1-100] | 42.9 | [32.1-54.1] | 52.5 | [42.3-62.5] | 97.3 | [85.8-99.9] |
| 25-+ | 283 | 11.3 | 81.3 | [63.6-92.8] | 72.9 | [67.0-78.3] | 27.7 | [18.9-37.8] | 96.8 | [93.2-98.8] |
| P-value |  |  | <0.001^(†)^ | <0.001 ^(†)^ | <0.001^(±)^ | 0.77^(†)^ |  |  |  |  |
